# Clinical Value of Routine Follow-Up Radiographs in Total Joint Arthroplasty

**DOI:** 10.7759/cureus.73990

**Published:** 2024-11-19

**Authors:** Kimberly V Ponsworno, Ali Rajab, Robert Keehan, Riaz Ahmad

**Affiliations:** 1 Trauma and Orthopaedics, Bristol Royal Infirmary, Bristol, GBR; 2 Trauma and Orthopaedics, University Hospitals Bristol and Weston NHS Foundation Trust, Weston-super-Mare, GBR

**Keywords:** clinical value, radiographic findings, routine follow-up, total hip arthroplasty, total joint arthroplasty, total knee arthroplasty

## Abstract

Background: Performing routine radiographs after total joint arthroplasty (TJA) in post-operative follow-up, typically at four weeks and 12 months, in addition to baseline radiographs obtained immediately post-operatively, is common practice in many institutions. Despite research indicating it may not alter management, it is associated with substantial financial, resource, and time costs. This study aimed to assess the impact of routine radiographs on the management of TJA patients in a UK district general hospital.

Method: This retrospective observational study included patients who underwent total knee arthroplasty (TKA) or total hip arthroplasty (THA) between September 2019 and December 2020. Patient data, including demographics, surgery details, and follow-up outcomes, were extracted from electronic medical records. Follow-up visits were categorized as four-week and 12-month post-surgery intervals, allowing for variability in timing due to COVID-19-related disruptions. Radiographic assessments, including requests, reports, and findings from clinic letters, were reviewed to determine any radiological abnormalities or changes in management. Descriptive statistics were applied to evaluate the frequency and context of routine versus unplanned radiographs, providing insights into post-operative care patterns.

Results: A total of 173 TJA patients met the inclusion criteria, with 54 exclusions due to lack of follow-up. A total of 56 patients (32%) had routine radiographs within the one-year follow-up period. No radiological abnormalities were detected on these, and none of the patients returned to the theatre. Of the 24 patients who presented with acute clinical concerns and had unplanned radiographs, eight (33%) required a return to theatre.

Conclusion: Routine follow-up radiographs in our study did not reveal any significant abnormalities nor did they result in changes to patient management, indicating a lack of clinical utility. Given that these radiographs impose considerable financial and resource burdens, their necessity is questionable. Based on the National Health Service (NHS) tariff costs, the potential savings from discontinuing routine radiographs in our cohort amounted to £3,129 annually. Extrapolating this to the national level, with approximately 150,000 total knee and hip replacements performed each year in the UK, suggests that substantial costs could be avoided.

## Introduction

Routine radiographs (RRs) are commonly performed in follow-up clinics after total knee arthroplasty (TKA) and total hip arthroplasty (THA), typically at four weeks and 12 months post-operatively, even though there are no clinical concerns [1]. Existing literature shows variability in practice, with institutional norms often guiding their use [1,2]. Birir et al. highlighted the lack of established clinical guidelines regarding the timing or indications for post-operative radiographs [2]. However, this practice raises significant questions, as recent studies indicate that RRs may provide limited clinical value and often fail to alter management decisions [1,2].

It is important to distinguish RRs from baseline radiographs, which are taken immediately after surgery to assess implant positioning and detect early complications, and from unplanned radiographs, which are conducted in response to acute clinical issues like trauma, pain, or swelling. The financial costs, clinical time, staffing requirements, and radiation exposure associated with RRs have led to a re-evaluation of their necessity in routine follow-up.

This study aims to evaluate the impact of RRs on clinical outcomes and management decisions for patients who underwent TKA and THA at a UK district general hospital. We hypothesize that RRs do not influence patient management compared to cases without routine imaging. By providing empirical data, this study aims to contribute to ongoing studies regarding the appropriateness of routine follow-up imaging in total joint arthroplasty (TJA).

## Materials and methods

This retrospective study included adult patients (≥18 years) who underwent TKA or THA at a district general hospital between September 1, 2019, and December 31, 2020. Patient demographics and clinical data were extracted from electronic medical records.

Inclusion and exclusion criteria

The study population included all adults who underwent primary TKA or THA during the specified timeframe and attended clinic follow-up appointments. Patients who did not attend clinic follow-ups were excluded from the study due to a lack of clinical outcome data.

Follow-up protocol

All patients undergoing TJA were scheduled for routine follow-up at approximately four weeks and 12 months post-operatively. During the COVID-19 pandemic, while many elective follow-ups shifted to telemedicine, our hospital continued to conduct in-person appointments within 12 months for routine radiographs and evaluations to monitor post-surgical outcomes. This standardized approach enhances the generalizability of the findings to be consistent with TJA management practices in our institution. Additionally, unplanned emergency department visits related to the operated joint (e.g., due to trauma, pain, or swelling) were included in the study.

Radiograph classification

For this study, radiographs were categorized into three distinct types. Baseline radiographs were taken immediately following surgery to assess implant positioning and identify any early complications. Routine radiographs were those performed at scheduled follow-up appointments, typically around four weeks and 12 months post-operatively, even when patients showed no specific clinical concerns. Lastly, unplanned radiographs were obtained outside of scheduled follow-ups in response to acute clinical issues, such as trauma, pain, or swelling, that required urgent assessment. This classification allowed for a systematic analysis of radiograph use and its relevance to patient management.

Data collection and analysis

Radiograph requests were systematically reviewed and categorized according to the mentioned classification. For each patient, clinic letters were examined to identify any changes in management or subsequent surgical interventions. Abnormal findings, such as malalignment, loosening, fractures, and soft tissue swelling, were identified through a review of radiograph reports and consultant findings in corresponding clinic letters. The clinic letters provided context for patients' symptoms, allowing correlation between radiographic abnormalities noted by consultants and radiologists. Any changes in management requiring surgical intervention were highlighted in the clinic letters.

All data were organized into TKA, THA, and combined TJA groups. Descriptive statistics were used to analyse the data. This included calculating the frequency and percentage of routine versus unplanned radiographs, as well as identifying the proportion of abnormal findings among the different categories. Mean values with standard deviations were calculated for continuous variables such as age. Microsoft Excel (Microsoft Corporation, Redmond, WA) was used for the analysis.

## Results

**Figure 1:**
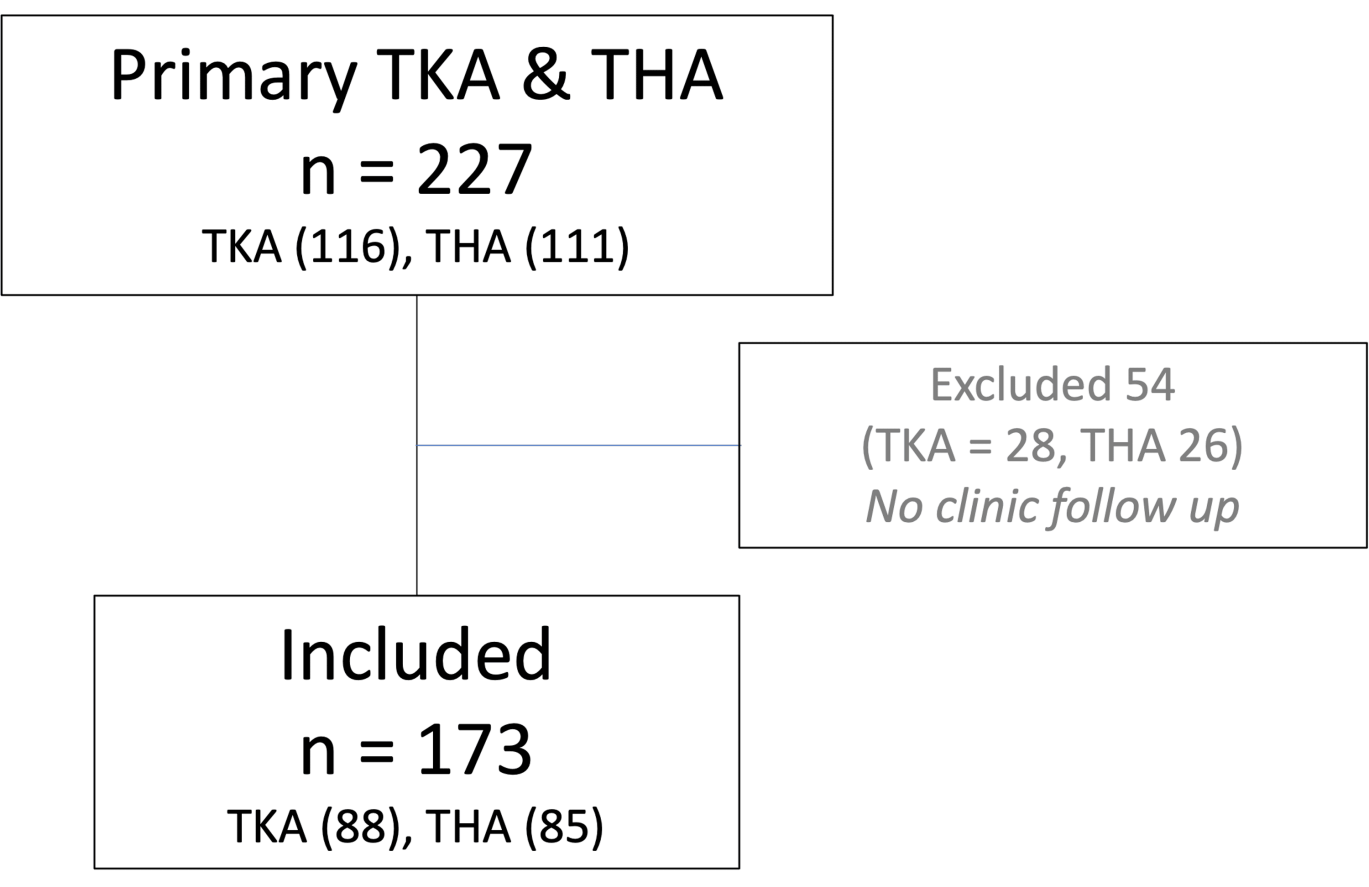
Patient inclusions and exclusions. TKA: total knee arthroplasty; THA: total hip arthroplasty.

A total of 227 TJA cases were reviewed for clinical data during the study period. A total of 173 patients (Figure 1) met the inclusion criteria. Of the patients, 54 were excluded due to lack of follow-up, attributed in all cases to changes in follow-up practice in response to the COVID-19 pandemic. The included cases comprised 88 TKAs (51%) and 85 THAs (49%) (Figure 1). Patient demographics are summarized in Table 1.

**Table 1:** Patient demographics. TKA: total knee arthroplasty; THA: total hip arthroplasty; TJA: total joint arthroplasty.

Patient factors	TKA (n = 88)	THA (n = 85)	TJA (n = 173)
Mean age (SD)	73.2 (9.3)	74.2 (11.3)	73.7 (10.4)
Sex			
Female	63 (71.6%)	58 (68.2%)	121 (69.9%)
Male	25 (28.4%)	27 (31.8%)	52 (30.1%)

Radiographic findings were analysed for all included patients (Table 2). RRs were obtained in 56 patients (31 (35%) TKA and 25 (29%) THA), none of which revealed significant abnormalities. None of these 56 patients required further clinic follow-up, and they did not receive any ongoing management or intervention related to their surgical outcomes.

**Table 2:** Patient follow-up and ED attendance with radiographic data. TKA: total knee arthroplasty; THA: total hip arthroplasty; TJA: total joint arthroplasty; XR: X-ray.

	TKA (n = 88)	THA (n = 85)	TJA (n = 173)
ED visit			
Attendance	14	10	24
Unplanned XR performed	14	10	24
Abnormal XR findings	6	2	8
4 to 6 weeks follow-up			
Attendance	69	64	133
Routine XR performed	28	21	49
Abnormal XR findings	0	0	0
12-month follow-up			
Attendance	5	11	16
Routine XR performed	3	4	7
Abnormal XR findings	0	0	0

A total of 24 patients (14%) presented outside of their planned clinic dates and underwent unplanned radiographs due to clinical concerns such as trauma, pain, or swelling. Of these, eight patients (33%) required a return to the operating theatre (Table 3). The remaining 16 patients (67%) did not undergo surgical intervention but received additional analgesia and physiotherapy.

**Table 3:** Surgical intervention in non-routine radiographs. TKA: total knee arthroplasty; THA: total hip arthroplasty; DAIR: debridement antibiotics and implant retention; MUA: manipulation under anaesthesia; PJI: prosthetic joint infection; ORIF: open reduction and internal fixation.

Procedure	TKA (n = 6)	Procedure	THA (n = 2)
DAIR	3	Revision for PJI	1
Excision of ossicle	1		
MUA & arthroscopic synovectomy	1	ORIF periprosthetic fracture	1
Revision long stem hinge TKA	1		

The total costs, time, and radiation exposure associated with all radiographs for the study population are summarized in Table 4.

**Table 4:** Cost, time (estimates), and radiation exposure of one knee and hip X-ray (XR). 1. Cost data: 2020/21 National Tariff Payment System (https://www.england.nhs.uk/wp-content/uploads/2021/02/20-21_National-Tariff-Payment-System.pdf). 2. Time: Time estimates were provided by our radiology department. 3. Radiation exposure data: Medical radiation: patient dose information (https://www.gov.uk/government/publications/medical-radiation-patient-doses/patient-dose-information-guidance#x-ray-examinations).

Factor	Knee	Hip
2-view XR (£)	59	52
XR request (min)	3	3
XR department (min)	15	20
Radiation (mSv)	<0.01	0.3

## Discussion

The utility of RRs following TJA remains a contentious issue among orthopaedic surgeons [2]. Generally, RRs are performed to identify early signs of failure that might require intervention. However, a prospective cohort study in the USA of 56 TJA patients found that RRs had a limited impact on patient management [3]. Additionally, Kingsbury et al. demonstrated that routine follow-up within a one- to 10-year period for non-complex total knee replacement and total hip replacement can be safely discontinued, including both clinical and radiographic evaluations, as long as patients have access to orthopaedic review if symptoms arise [4]. These findings suggest that similar outcomes are observed both in the UK and internationally.

Our review of nearly 200 patients from a district general hospital also revealed no clinically significant findings on RRs, leading to the discharge of all patients from follow-up without additional investigations or management changes. This suggests that RRs within the first 12 months may not be necessary. In contrast, some patients who had unplanned radiographs in response to symptoms such as trauma, pain, or swelling, did go on to have surgical interventions. This supports the principle that radiographs should be based on clinical need and evaluated on a case-by-case basis, rather than being conducted routinely.

Similar studies support our findings. Hart et al. reviewed nearly 900 TJA RRs at a tertiary institution and found a low incidence of abnormal findings that did not alter clinical management for up to one year [2]. Single-joint studies by Birir et al. (TKA) and Christensen and Folkmar (THA) have similarly suggested omitting RRs from outpatient follow-up [2,5]. Röder et al. analysed follow-up data from 18,486 THA performed between 1967 and 2001 to evaluate the effectiveness of clinical methods in diagnosing prosthetic component loosening. They determined that routine clinical and radiological follow-up for asymptomatic patients was unnecessary in the first five to six years [6]. Additionally, King et al. found no clinical difference between TKA patients who missed clinical and radiological follow-ups from six months to five years post-operation and those who attended routine evaluations [7].

Reducing unnecessary radiation exposure is essential. Although standard knee and hip radiographs incur only minimal radiation and are not considered to be associated with risks [8], reducing radiation exposure to zero by not performing a radiograph at all could be less risky [9].

Recent efforts to reduce NHS costs have highlighted the need for cost-effective practices. The financial implications of our findings are significant. Our results indicate that eliminating routine RRs for TJA could reduce costs, as these radiographs did not prompt changes in clinical management but accounted for significant follow-up time and expenses. In addition, eliminating these could save £3,129 annually for our study’s sample. The National Institute for Health and Care Excellence (NICE) estimates the total cost for the first 12 months of outpatient care, including NHS-provided aids, adaptations, and medications, to be £394 for a THA [10].

With nearly 150,000 TJAs performed annually in the UK, foregoing RRs could result in substantial savings [11]. Thus, we recommend forgoing routine RRs and focusing resources on symptomatic patients who are more likely to require further management. This approach could reduce unnecessary orthopaedic appointments, radiation hazards, and radiology bookings, reallocating resources more effectively. In response to escalating healthcare costs and NHS budgetary constraints, practices such as this, which can be demonstrated to be cost-effective, should be adopted [12,13].

Our study has several limitations. The sample size was relatively small, with a relatively large number of exclusions, both mainly due to the COVID-19 pandemic. The study was conducted at a single district general hospital, which may limit generalizability. Additionally, the absence of a control group or randomization affects the robustness of the findings. Future research with larger sample sizes, standardized radiographic criteria, and prospective data could provide more definitive conclusions.

## Conclusions

Our study presents evidence that challenges the traditional use of routine post-operative radiographs following TJA. The findings show that RRs have minimal impact on clinical management and incur significant costs, highlighting the need to reconsider current follow-up protocols. Our results suggest that routine RRs may be safely discontinued, with radiographs reserved for cases where clinical indications, such as patient-reported symptoms, warrant further investigation. Although limited to a single setting, this study underscores the potential benefit of multicentre studies to build a more robust evidence base on the necessity and effectiveness of routine radiographs. Such an approach could optimize resource allocation and improve patient outcomes across the healthcare system.
